# *Rhaponticum uniflorum* and *Serratula centauroides* Extracts Attenuate Emotional Injury in Acute and Chronic Emotional Stress

**DOI:** 10.3390/ph14111186

**Published:** 2021-11-19

**Authors:** Larisa N. Shantanova, Daniil N. Olennikov, Irinchey E. Matkhanov, Sergey M. Gulyaev, Anyuta A. Toropova, Irina G. Nikolaeva, Sergey M. Nikolaev

**Affiliations:** 1 Institute of General and Experimental Biology, Siberian Branch of Russian Academy of Science, Sakhyanovoy Str. 6, 670047 Ulan-Ude, Russia; olennikovdn@mail.ru (D.N.O.); s-gulyaev@inbox.ru (S.M.G.); anyuta-tor@mail.ru (A.A.T.); i-nik@mail.ru (I.G.N.); smnikolaev@mail.ru (S.M.N.); 2 Institute of Medicine, Buryat State University, Oktyabrskaya Str.36a, 670002 Ulan-Ude, Russia; matkhanov1965@mail.ru

**Keywords:** *Rhaponticum uniflorum*, *Serratula centauroides*, ecdysteroids, caffeoylquinic acids, emotional stress, depression, neuroprotection, antioxidant activity

## Abstract

In modern life, the use of plant stress-protectors has taken on particular significance due to the wide distribution of neurosis-like and neurotic diseases caused by neuroendocrine-immune system imbalance. Special attention has been paid to the plants containing ecdysteroids, i.e., hormone-like bioactive substances with high adaptogenic activity. The article deals with the study of bioactivity of two plant extracts as *Rhaponticum uniflorum* (L.) DC. and *Serratula centauroides* L. with a high content of ecdysteroids and phenolic compounds. The models of acute and chronic emotional stress in white rats were used to estimate the stress-protective activity of *R. uniflorum* and *S. centauroides* extracts. Both extracts showed the stress-protective effect via inhibiting the development of signs induced by single and long-term effects of stress factors. In acute stress, the development of Selye's triad signs was less pronounced against the background of the plant remedies introduction. In chronic stress, the extracts prevented the development of anxiety-depressive syndrome. Besides, *R. uniflorum* and *S. centauroides* extracts banned the development of stress-induced injuries in the brain cortex and had a neuroprotective effect on ischemia against chronic stress. The stress-protective effects of both plant extracts were based on a decrease of hyperactivation of the central stress-promoting systems (sympathoadrenal, hypothalamic-pituitary-adrenal) due to their GABA-mimetic effects. Peripheral mechanisms were connected with the inhibition of free radical oxidation processes and with an increase in the endogenous antioxidant system activity. Thus, *R. uniflorum* and *S. centauroides* extracts have a high potential to increase non-specific body resistance against acute and chronic emotional stress effects.

## 1. Introduction

Plant adaptogens are effective remedies to increase the non-specific resistance of the body to stress factors [1]. In recent decades, their use has taken on particular significance due to the widespread of neurosis-like and neurotic states in practically healthy persons caused by neuroendocrine-immune system imbalance [2,3]. In the COVID-19 pandemic conditions, borderline psycho-emotional disorders resulting in the development of stress-induced pathological states and negative disease outcomes were registered in 60–70% of the adult population around the world [4,5].

In this connection, the search and development of new plant adaptogenic remedies are now becoming ever more relevant, because plant adaptogens are complexes of bioactive substances structurally similar to endogenous regulatory compounds of the body and can ameliorate the functions of the neuroendocrine-immune system [6,7,8].

Special attention has been paid to the plants containing ecdysteroids, i.e., hormone-like bioactive substances, possessing a wide spectrum of pharmacological properties and also known as adaptogens [9,10]. Ecdysteroids are very common in plants from sections Polypodiophyta, Pinophyta and Magnoliophyta. Nevertheless, their quantity is really small which is only a tenth to hundreds of a percent that makes it difficult to use them as the ecdysteroid source. Only a few species including *Rhaponticum uniflorum* and *Serratula centauroides* contain more than 1 percent of ecdysteroids [11].

Two famous species with a high content of ecdysteroids are *Rhaponticum carthamoides* (Wild) Iljin and *Serratula coronata* L., both from the Asteraceae family, used to prepare officinal 20-hydroxyecdysone-containing preparations as Leuzea liquid extract, Ecdysten and Serpisten. The study of closely related species such as Asian plants *Rhaponticum uniflorum* (L.) DC. and *Serratula centauroides* L. is a promising direction for searching the new sources of ecdysteroids. These plants grow in Siberia and the Far East as well as in Northern Mongolia, North-Eastern China and Korea. The decoctions from these plants are used in the practice of East Asian traditional medicine [12,13].

Ecdysteroids in the plant were first revealed in the early 1990s and by now its ecdysteroid profile includes more than 30 individual compounds, among them, 20-hydroxyecdysone and ajugasterone derivatives are common. Some other bioactive substances have been found in *R. uniflorum* as flavonoids, di- and triterpenoids, sesquiterpenes, organic acids, lignans, essential oils, polysaccharides, amino acids, vitamins and fatty acids [14,15,16]. The pharmacological activity of *R. uniflorum* was shown mainly for the root extracts, decoction and solvent fractions. It has been established that *R. uniflorum* root extracts prevent aging and have anti-tumour, anti-inflammatory, antioxidant and immune-modulating activity [13,17]. The *S. centauroides* herb contains ecdysteroids with the highest concentration in the leaves (20.64 mg/kg) and stems (16.20 mg/kg). 20-Hydroxyecdysone is the basic compound in all organs [15,18]. Among other compounds, this plant also contains phenolic compounds, amino acids, fatty and organic acids and essential oils. Decoction of *S. centauroides* herb demonstrated hemostatic, anabolic, anti-hypoxic, nootropic and anxiolytic potential [19].

The present study aimed to estimate the stress-protective activity of two plant extracts from *Rhaponticum uniflorum* and *Serratula centauroides* in acute and chronic emotional stress.

## 2. Results

### 2.1. HPLC-DAD-ESI-MS Profiles of Rhaponticum uniflorum and Serratula centauroides Extracts

**Figure 1:**
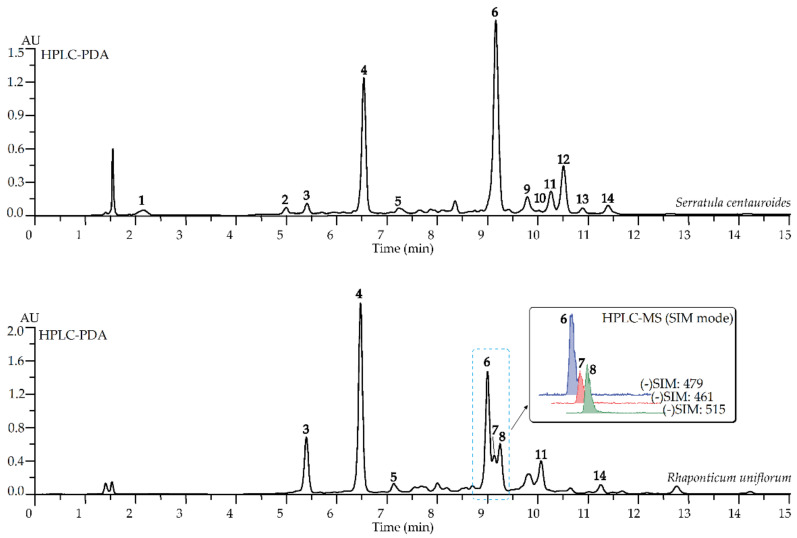
High-Performance Liquid Chromatography with Photodiode Array Detection (HPLC-PDA) chromatograms (λ 254 nm) and High-Performance Liquid Chromatography with Mass Spectrometric Detection (HPLC-MS) in Selective Ione Monitoring (SIM mode) chromatograms of total extracts of *Rhaponticum uniflorum* and *Serratula centauroides* herbs. Compounds are numbered as listed in Table 1.

Chromatographic study of *Rhaponticum uniflorum* and *Serratula centauroides* extracts by high-performance liquid chromatography with photodiode array and electrospray triple quadrupole mass detection (HPLC-PAD-ESI-tQ-MS) showed the presence of eight and twelve compounds, respectively (Table 1, Figure 1). Three basic compounds of *R. uniflorum* extracts were 5-*O*-caffeoylquinic acid, 4-*O*-caffeoylquinic acid and 20-hydroxyecdysone with the level of content at 63.59, 25.11 and 17.83 mg/g, respectively, as well in *S. centauroides* extract the major compounds were 5-*O*-caffeoylquinic acid (40.85 mg/g), 20-hydroxyecdysone (27.43 mg/g) and apigenin-7-*O*-glucuronide (18.27 mg/g) (Table 1). The remaining identified compounds were mainly di-*O*-caffeoylquinic acids and *O*-glycosylflavones found previously in herb of *R. uniflorum* [15,16,18,19].

### 2.2. Acute Emotional Stress

The study of the stress-protective activity of *R. uniflorum* and *S. centauroides* extracts in an acute emotional stress model showed that preventive 7-day introduction at a dose of 100 mg/kg was followed by the decrease in Selye’s triad manifestations and the intensity of free radical oxidation in white rats (Table 2). Table 2 shows that 18-h emotional stress resulted in the development of a specific pattern of stress injuries in inner organs: hypertrophy of the adrenal glands, involution of immune organs and induction of free radical oxidation processes. The preventive introduction of the tested extracts limited the development of stress-reaction signs: the mass of immune organs and adrenal glands in rats receiving the extract of *S. centauroides* was similar to that of intact rats. Against the background of *R. uniflorum* administration, hypertrophy of the adrenal glands was noted, but the mass of immune organs fell within the physiological range.

Additionally, the extracts from *R. uniflorum* and *S. centauroides* prevented the development of rough defects in the gastric mucosa of rats and decreased the number of erosions and ulcers by 33 and 66%, respectively, compared with a control group. The adaptogenic effect of the tested extracts was similar to that of the reference preparation *R. carthamoides* extract.

Against the acute emotional stress, significant depression of the endogenous antioxidant system was noted along with the consequential induction of free radical oxidation processes and accumulation of peroxidation products. 

Preventive introduction of the extracts from *R. uniflorum* and *S. centauroides* promoted the inhibition of free radical oxidation as indicated by the decrease of thiobarbituric acid reactive substances 30 and 50% respectively as compared to the control. The inhibitory effect of *R. uniflorum* and *S. centauroides* was due to antioxidant system activation, as indicated by the 3.0 and 3.5-fold increases in the concentration of reduced glutathione, respectively, the 25% increase in catalase activity and the 57% and 82% increases in superoxide dismutase activity, respectively, as compared to the control rats. 

The inhibition of free radical oxidation processes was due to the capacity of the extracts to render a direct antiradical effect that was indicated by in vitro tests. Particularly, we studied 2,2-diphenyl-1-picrylhydrazyl radical (DPPH^•^) scavenging activity, 2,2′-azino-bis(3-ethylbenzothiazoline-6-sulfonic acid cation-radical (ABTS^•+^) scavenging activity, superoxide-anion scavenging activity and Fe^2+^-chelating activity (Table 3).

The radical-scavenging activity of *R. uniflorum* and *S. centauroides* against DPPH^•^ was high, with IC_50_ 22.80 μg/mL and 30.12 μg/mL, respectively. The ABTS^•+^ radical cation-scavenging activity was also high, with IC_50_ 15.50 μg/mL and 25.14 μg/mL, respectively. The same parameters found for ascorbic acid were 4.84 μg/mL (DPPH^•^) and 9.11 μg/mL (ABTS). The superoxide-anion scavenging activity of *R. uniflorum* and *S. centauroides* was IC_50_ 55.40 μg/mL and 77.31, higher than the activity of ascorbic acid (101.10 μg/mL). Besides, the *R. uniflorum* and *S. centauroides* extracts had chelating action on Fe^2+^ ions, with IC_50_ 546.51 and 819.20 μg/mL, respectively, thus inhibiting free radical oxidation process intensity.

The tested plant extracts had this effect not only on the peripheral antioxidant system but also on the central stress-limiting systems of the body, thereby inhibiting their hyperactivation in acute emotional stress (Table 4). 

The data presented in Table 4 show that the preventive introduction of *R. uniflorum* and *S. centauroides* extracts was followed by a decrease in the activity of the trigger chain of the stress-reaction, i.e., the sympathoadrenal system, as indicated by a 30% decrease in the adrenaline concentration, whereas noradrenalin was decreased by 25% and 13%, respectively, as compared to control animals. Moreover, under the influence of *R. uniflorum* and *S. centauroides* extracts, there is a decrease in adrenocorticotropic hormone content by 40% and 50%, respectively, and in corticosterone levels by 24% and 50%, respectively, as compared to the control. The content of aldosterone under the influence of the *R. uniflorum* extract decreased by 24% but the *S. centauroides* extract did not influence this index. These data indicate the inhibition of hypothalamic-pituitary-adrenal system activity under acute emotional stress. 

Finally, the efficiency of *R. uniflorum* and *S. centauroides* extracts in acute emotional stress was comparable with that of the reference preparation (*R. carthamoides* extract) and the antioxidant activity in tested preparations was higher than in the reference preparation. 

### 2.3. Chronic Emotional Stress

The long-term influence of emotional stress factors is followed by the development of a chronic stress pattern in white rats, as indicated by specific changes in target organs, i.e., hypertrophy of the adrenal glands and involution of immune-competent organs as well as the development of an anxiodepressive state with signs of anhedonia, behavioural disorders in animals and neurodistrophic changes in the cerebral cortex.

**Figure 2:**
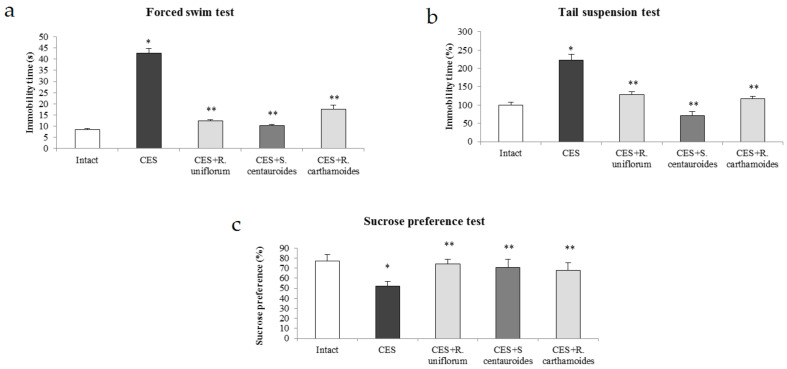
Antidepressive effects of *R. uniflorum* and *S. centauroides* extracts in the forced swim (**a**), tail suspension (**b**) and sucrose preference tests (**c**) in the model of chronic emotional stress (CES). Data represent as mean ± S.E.M. *—*p* < 0.05 vs. intact group; **—*p* < 0.05 vs. CES group.

The development of an anxiodepressive state is indicated by a significant increase in the immobility period in rats of the control group in the forced swimming and tail suspension tests (Figure 2). Against the background of chronic emotional stress, the introduction of *R. uniflorum* and *S. centauroides* extracts at a dose of 100 mg/kg rendered a significant anti-depressive effect: the immobility time in animals of the experimental groups was reduced by 2–4 times on average as compared to the control group.

Anhedonia is a specific sign of a depressive state, as indicated by the results of the sucrose preference test. The rats in the control group were found to reduce their consumption of sucrose solution by 33% as compared to intact animals (Figure 2). In contrast, the animals of the experimental groups, which received the tested extracts, consumed sucrose 1.4 times more than the control animals. This index did not differ from that of the intact animals. The antidepressive effects of the tested extracts were similar to those of the reference preparation *R. carthamoides* extract.

Chronic stress in white rats was also characterised by changes in behavioural activity, indicative of a high level of anxiety: in control animals, the time spent in the open arms of the elevated plus maze and the number of entries into them were significantly less than in intact rats. Besides, the level of exploration activity decreased and was accompanied by vegetative disorders such as an increase in defecation (Table 5).

The introduction of the tested extracts against the background of chronic stress promoted high tolerance for stress and a low level of anxiety, inhibiting the fear of the open field and stimulating locomotor exploration activity in rats of the experimental groups. The number of entries into the open arms of elevated plus maze and the time spent in them was increased by 8- and 3-fold, respectively, in rats treated with *R. uniflorum* and in rats treated with *S. centauroides*, it was increased by 10- and 3.7-fold, respectively, as compared to the control. In the ‘open field’ test, the predominance of vertical activity over horizontal activity as well as the stimulation of hole exploratory behaviour was noted in rats of the experimental groups, suggesting the activation of exploration behaviour in animals. Against the background of *R. uniflorum* and *S. centauroides* extract administration, the vertical activity was increased by 4 times on average and the index of hole exploratory behaviour (estimated by the number of times rats peeked into the holes) was increased by 3.8 and 5.0 times as compared to the control. A reliably significant decrease in the number of boli (by 32%) was observed under the influence of *S. centauroides* as compared to the control rats.

**Figure 3:**
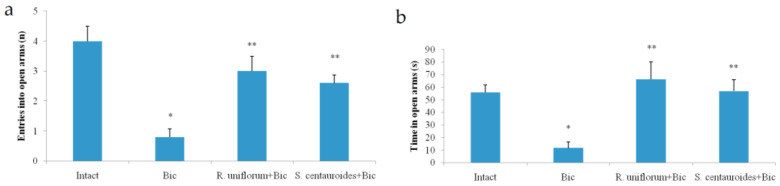
Entries into open arms (**a**) and time in open arms (**b**) in elevated plus maze test after *R. uniflorum* and *S. centauroides* extracts application against bicuculline (Bic) introduction. Data represent as mean ± S.E.M. *—*p* < 0.05 vs. intact group; **—*p* < 0.05 vs. Bic group.

The mechanisms of the anxiolytic effect of *R. uniflorum* and *S. centauroides* extracts were studied in an independent series of experiments with the use of GABA-A receptors inhibitors bicuculline and picrotoxin. The *R. uniflorum* and *S. centauroides* extracts demonstrated the anxiolytic effect against the background of Bic introduction: the time spent in the open arms of the elevated plus maze was 5.7 and 4.9 times longer, respectively, and the number of entries was 3.7 and 3.2 times more than in rats receiving bicuculline (*p* < 0.05) (Figure 3). The data show that the tested extracts demonstrate antagonism against Bic, which is a GABA-A receptor competitive inhibitor.

**Figure 4:**
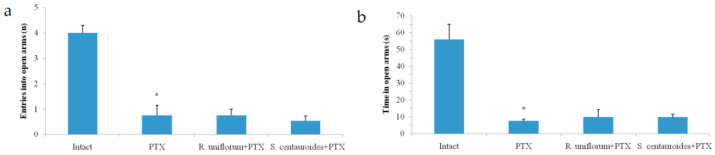
Entries into open arms (**a**) and time in open arms (**b**) in elevated plus maze test after *R. uniflorum* and *S. centauroides* extracts application against picrotoxin (PTX) introduction. Data represent as mean ± S.E.M. *—*p* < 0.05 vs. intact group.

However, the anxiolytic effect of the *R. uniflorum* and *S. centauroides* extracts was quenched by picrotoxin introduction: there were no differences between the indices of the experimental groups and picrotoxin group (Figure 4) indicative of the absence of competitive interactions with picrotoxin due to different binding sites on the GABA-A receptor. Picrotoxin is known to be a non-competitive GABA-A receptor antagonist via quenching chloride channels and hence GABA-ergic currents [20]. This may explain the levelling of the GABA-ergic influence of the extracts by this antagonist.

**Figure 5:**
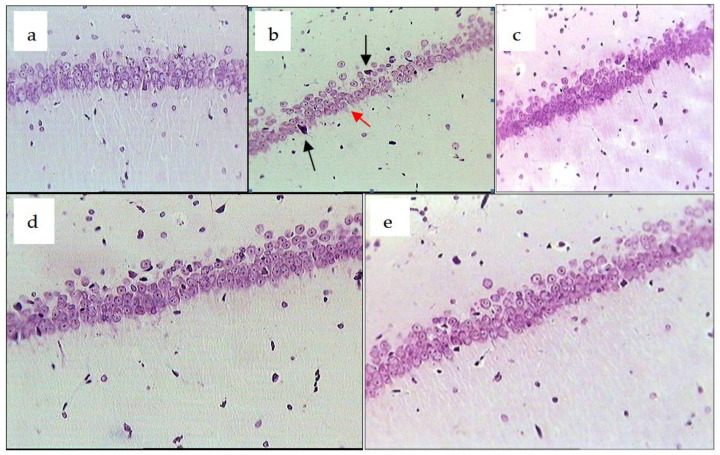
Dorsal hippocampus (CA1, ipsilateral side) in rats exposed to chronic emotional stress (CES). Experimental groups: intact, no CES (**a**); CES, decreased neuron density of the pyramidal layer (**b**); black arrow–necrosis of neuron, red arrow–autolysis; CES + *R. uniflorum* (**c**); CES *+ S. centauroides* (**d**); CES + *R. carthamoides* (**e**). Stained with cresyl-violet. Magnification × 400.

The pathomorphological study showed that chronic emotional stress in rats resulted in the disturbance of the dorsal hippocampus cytoarchitecture (Figure 5).

In animals of the control group, the disintegration of the pyramid level, predominantly in the CA1 area, was noted along with neuronal injury in the form of dystrophic changes such as cytoplasmic vacuolisation with the lack of Nissl substance and changes in the form of the cell and nucleus which are often off-centred. Neurons with signs of autolysis, necrosis and/or apoptosis were found less often (Figure 5b). In rats treated with the *R. uniflorum* and *S. centauroides* extracts and *R. carthamoides* (reference preparation) against the background of chronic emotional stress, the pyramid level remained well-ordered (Figure 5c–e). In many cases, the neurons had a normal structure: round form of the body and nucleus and Nissl substance in the cytoplasm. Dystrophic changes were reversible in nature (without rough injuries in the cytoplasm and nucleus) and partial chromatolysis was noted only in a small number of neurons.

**Figure 6:**
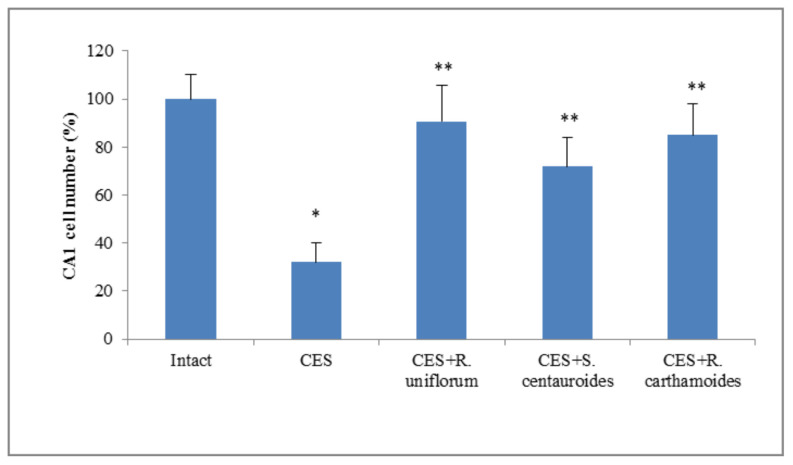
The effect of *R. uniflorum* and *S. centauroides* extracts on the number of viable neurons in the dorsal hippocampus (CA1) in the rats exposed to chronic emotional stress (CES). Data represent as mean ± S.E.M.*—*p* < 0.05 vs. intact group; **—*p* < 0.05 vs. CES group.

The number of normal neurons in the dorsal hippocampus of the rats exposed to stress was 68% less than in the intact animals as a result of destructive cell changes and processes of restructuring of the pyramid level after stress (Figure 6). 

In rats treated with the *R. uniflorum* and *S. centauroides* extracts, the number of normal neurons in the dorsal area of the hippocampus was 58 and 40% higher, respectively than in the control rats. 

**Figure 7:**
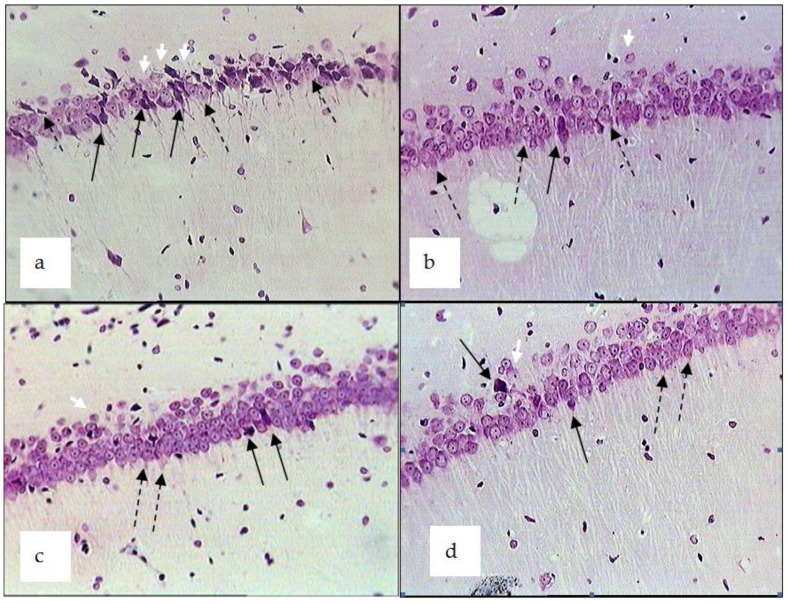
Dorsal hippocampus CA1 (ipsilateral side) in rats subjected to chronic emotional stress (CES) and ischemia (**I**). Experimental groups: CES + I (**a**); CES + I + *R. uniflorum* (**b**); CES + I + *S. centauroides* (**c**); CES + I + *R. carthamoides* (**d**). Solid black arrow—hyperchromic shrunken neurons; white arrow—cytolysis; dotted arrow—dystrophic changes to neurons; disintegration of the pyramidal layer. Stained with cresyl violet. Magnification ×400.

Unilateral left occlusion of the rats’ common carotid artery performed against the background of chronic stress caused more severe ischemic injuries in the neurons of the brain, mainly on the ipsilateral side. In the dorsal area of hippocampus CA1 (ipsilateral side) of the control rats (chronic emotional stress + ischemia), neuronal injuries were noted in the form of hyperchromia, severe dystrophy with vacuolisation and changes in the form of cells and nuclei (Figure 7). The morphometric study showed that, in the rats exposed to chronic emotional stress and ischemia, the proportion of hyperchromatic shrunken neurons and neurons with dystrophy was 64% and 16%, respectively, as compared to the indices in the sham-operated rats (Table 6). On the contralateral side, the proportion of hyperchromatic and dystrophic neurons was 21% and 17% respectively. The proportion of normal neurons in the left and right brain was 20% and 60%, respectively.

The administration of the tested extracts to the rats against a background of chronic stress and brain ischemia demonstrated a neuroprotective effect: on the ipsilateral side, the number of hyperchromatic neurons was 30% lower in rats treated with *R. uniflorum* and *S. centauroides* extracts and the number of normal neurons was 44% and 84% higher, respectively, as compared to the control rats (chronic emotional stress + ischemia). On the contralateral side, the differences in the above indices of the experimental groups were lower than the indices in the control. On the whole, the indices of the stress-protective and neuroprotective effects of both tested extracts against the background of stress and brain ischemia were comparable with the indices of the preparation of comparison, i.e., *R. carthamoides* extract.

## 3. Discussion

The administration of *R. uniflorum* and *S. centauroides* extracts at the experimental-therapeutic dose had a stress-protective effect, as indicated by the amelioration of the neuro-endocrine system imbalance in white rats exposed to acute and chronic emotional stress. It seems clear that bioactive substances of plant adaptogenes, being the natural bio-regulatory compounds, promote the more effective functioning of bodily adaptation under stress conditions. Diminishing the negative influence of hyperergic injuries at the stage of the anxiety reaction reinforces restorative metabolic processes at the resistance stage and eliminates or inhibits the development of the exhaustion stage [1,21].

The stress-protective effects of *R. uniflorum* and *S. centauroides* extracts are connected with their inhibiting influence on basic stress-associated mechanisms functioning at different levels of homeostatic: regulation. Hyperactivation of the sympathoadrenal system and induction of free radical oxidation processes are known to be the triggering mechanisms of the stress reaction [1,22,23,24]. The decrease in stress hormone concentrations (adrenaline, noradrenaline, adrenocorticotropic hormone and corticosterone) under the influence of *R. uniflorum* and *S. centauroides* extracts in emotional stress indicates the restricting hyperactivation of the sympathoadrenal and hypothalamic-pituitary-adrenal systems. Constituent tetracyclic triterpenoids, phenylpropanoids etc. which structurally resemble catecholamines and glucocorticoids [6,25], interact with these hormone receptors, thereby decreasing their sensitivity to stress factors.

Restricting the hyperactivation of stress-promoting systems under the influence of *R. uniflorum* and *S. centauroides* extracts also seems to be connected with the GABA-mimetic effects of ecdysteroids, which were verified experimentally with the use of a competitive antagonist of GABA-A receptors, bicuculline. Reportedly, 20-hydroxyecdysone, a dominant compound of *R. uniflorum* and *S. centauroides* extracts, has an affinity for GABA-A-receptors, promoting GABA-ergic currents in experiments on rat neuron cultures [26]. 

The development of chronic emotional stress in our experiments was followed by neuron injuries in the CA1 area of the dorsal hippocampus, in good agreement with literature data [27]. The neurons of the given area are known to be extremely vulnerable to stress due to the high density of glutamate and glucocorticoid receptors [28]. Unilateral occlusion of the rats’ common carotid artery performed against the background of chronic stress caused even more pronounced neuronal injuries in the brain, mainly on the ipsilateral side. In general, the administration of the tested extracts had a neuroprotective effect with regard to stress and ischemic impacts.

Since oxidative stress is known to be the basis of stress and ischemic injuries, including to the structures of the brain [29], we may suggest that the molecular-cellular mechanisms of anti-stress and neuroprotective effects of *R. uniflorum* and *S. centauroides* extracts are due to free radical oxidation process inhibition and the activation of the endogenous antioxidant system, which directly protects cell membrane structures from free radicals under stress. Our experiments demonstrate that the administration of *R. uniflorum* and *S. centauroides* extracts against a background of emotional stress had a marked antioxidant effect, indicated by the decrease in the concentration of TBARS, the increase in the concentration of reduced glutathione and the improved activity of superoxide dismutase and catalase. The in vitro tests showed that the antioxidant properties of *R. uniflorum* and *S. centauroides* extracts are due to their direct antiradical effect against DPPH^•^, ABTS^•+^, O^2•-^ radicals and Fe^2+^ ion chelates. 

The antioxidant properties of *R. uniflorum* and *S. centauroides* extracts are connected with the availability of the constituent dominating polyphenolic compounds such as 4-*O*-caffeoylquinic acid (content in *R. uniflorum* and *S. centauroides* 25.11 ± 0.50 and 4.19 ± 0.08 mg/g respectively), 5-*O*-caffeoylquinic acid (content: 63.59 ± 1.27 and 40.85 ± 0.82 mg/g); and apigenin-7-*O*-glucuronide (content: 16.93 ± 0.37 and 18.27 ± 0.36 mg/g). Besides their antioxidant activity is due to the high content of ecdysteroids, the chief of which is 20-hydroxyecdysone (content: 17.83 ± 0.35 and 27.43 ± 0.55 mg/g). The given compounds reportedly stimulate ATP synthesis, limit the generation of free radicals, promote the activation of the antioxidant protection of the body and inhibit free radical oxidation processes and the glutamate-calcium cascade that induces the development of stress-induced injuries [26,29,30]. 

Thus, the anti-stress effect of *R. uniflorum* and *S. centauroides* extracts has a multi-target nature. These effects are due to the activation of both central and peripheral stress-limiting systems of the body, thus providing adaptation to conditions of acute and chronic emotional stress.

## 4. Materials and Methods

### 4.1. Preparation of R. uniflorum and S. centauroides Extracts

The dry extracts were derived from the herb of *R. uniflorum* and *S. centauroides*. The plant material was gathered during the flowering period of 2018–2019 in the forest-steppe zone of Russia's Trans-Baikal region, the Republic of Buryatia, Ivolginsky district, Klyuchi Village; N 51°40.262′, E 107°11.314′, h 783 m. To obtain the dry extracts, the milled and weighed sample of the *R. uniflorum* herb (100 g) was successively extracted with 60% ethanol and twice with 30% ethanol at 60 °C. The raw material-to-solvent ratio was 1:12. The first extraction time was 90 min; the second and third extraction time was 60 min. In the same way, the weighed sample of the *S. centauroides* herb (100 g) was successively extracted with 70%, 40% and 20% ethanol (the raw material-to-solvent ratio was 1:12). The first extraction time was 90 min and the second and third extraction time was 60 min each. The extracts obtained were filtered, concentrated in a rotary vacuum evaporator and dried in the vacuum oven. The yield of the *R. uniflorum* and *S. centauroides* extracts was 30.0 g and 37.2 g respectively. The liquid extract of *Rhaponticum carthamoides* roots (specification 20-hydroxyecdysone content 10 mg/100 mL) was purchased by Kamelia NPP (Moscow, Russia).

### 4.2. High-Performance Liquid Chromatography with Photodiode Array Detection and Electrospray Ionization Triple Quadrupole Mass Spectrometric Detection (HPLC-PDA-ESI-tQ-MS)

Chromatographic analysis of herbal extracts realised by high-performance liquid chromatography with photodiode array detection and electrospray ionization triple quadrupole mass spectrometric detection (HPLC-PDA-ESI-tQ-MS) technique using a liquid chromatograph LC-20 Prominence coupled with photodiode array detector SPD-M30A (wavelength range 200–600 nm) and triple-quadrupole mass spectrometer LCES 8050 (all Shimadzu, Columbia, MD, USA) and C18 column GLC Mastro (150 × 2.1 mm × 3 μm; Shimadzu, Kyoto, Japan). Two-eluent gradient elution was used for successful separation of compounds: column temperature 30 °C; eluents A, 0.5% HCOOH in water; eluent B, 0.5% HCOOH in MeCN; gradient program: 0–2 min 5–6% B, 2–9 min 6–11% B, 9–15 min 11–25% B, 15–20 min 25–55% B, 20–25 min 55–5% B. The injection volume was 1 μL and the elution flow was 100 μL/min. The UV-Vis spectra were registered in the spectral range of 200–600 nm. Mass spectrometric detection was performed both in negative and positive ESI mode and the temperature levels of ESI interface, desolvation line and heat block were 300 °C, 250 °C and 400 °C, respectively, and the flow of nebulising gas (N_2_), heating gas (air) and collision-induced dissociation gas (Ar) were 3 L/min, 10 L/min and 0.3 mL/min, respectively. The mass spectra were registered as 3 kV source voltage and collision energy +15–+25 eV in the positive mode and −15–35 eV in the negative mode by the scanning range of *m*/*z* 50–2000. LabSolution’s workstation software with the inner LC-MS library was used to manage the LC-MS system. The final identification of metabolites was done after an integrated analysis of retention time, ultraviolet and mass spectra with the reference samples and/or literature data. To prepare the sample solution, the total extract (100 mg) was ultrasonically dissolved in 25 mL of 50% acetonitrile, filtered through a 0.22-μm PTFE syringe and stored at 4 °C before analysis.

Quantification of compounds was done in chromatographic conditions as described above and the HPLC-MS data (total peak area;) was used for calculation. Fourteen metabolites were quantified and thirteen compounds including arbutin, 1-*O*-caffeoylquinic acid, 4-*O*-caffeoylquinic acid, 5-*O*-caffeoylquinic acid, 1,3-di-*O*-caffeoylquinic acid, 20-hydroxyecdysone, luteolin-7-*O*-glucuronide, 3,4-di-*O*-caffeoylquinic acid, 3,5-di-*O*-caffeoylquinic acid, apigenin-7-*O*-glucuronide, chrysoeriol-7-*O*-glucoside, 4,5-di-*O*-caffeoylquinic acid, 1,5-di-*O*-caffeoylquinic acid prepared after careful weighing (10 mg) and dissolution in the methanol-DMSO mixture (1:1) in volumetric flasks (10 mL). Inokosterone content was expressed as 20-hydroxyecdysone equivalents and chrysoeriol-7-*O*-glucuronide as chrysoeriol-7-*O*-glucoside. To build reference standard calibration curves, the stock solutions were diluted with methanol (1–100 µg/mL), chromatographed and total MS peak area data were used to plot “concentration–peak area” graphs. The validation criteria (correlation coefficients, *r*^2^; standard deviation, *S*_YX_; limits of detection, LOD; limits of quantification, LOQ; and linear ranges) were calculated as described previously [31] (Table 7). All analyses were carried out three times and the data were expressed as mean value ± standard deviation (S.D.). 

### 4.3. Laboratory Animals

The experiments were carried out on white male and female Wistar rats weighing 170–180 g. The animal care was compliant with the ‘Rules of Laboratory Practice’ (GLP) and the Order of the Russian Health Ministry ‘Rules of Laboratory Practice’ (no. 199N, 01.04.2016). The animals were maintained under the standard laboratory conditions of the certified vivarium at the Institute of General and Experimental Biology SB RAS (t—20–22 °C, humidity—no more than 50%, air exchange (air intake/outlet)—8:10, light conditions (day/night)—1:1) with free access to water. The rats were housed 8 per plastic cage. The experimental work followed the ‘European Convention for the protection of vertebrate animals used for experimental and other scientific purposes’ ETS no. 123 dated 18.03.1986 (Strasburg, 1986). The animals were removed from experiments by instantaneous decapitation under brief ether anaesthesia. The design and protocol of research works were approved by the Ethics Committee at the Institute of General and Experimental Biology SB RAS (protocol no. 1, 15.01.2018). The animals were randomised with due account of sex, age, weight and resistance to stress. Because rats with high and low resistance to stress were characterised by different behavioural patterns, we selected animals with average stress resistance. In preliminary dose-effect relationship experiments, we studied the dose range 25, 50, 100, 150 and 200 mg/kg. The adaptogenic effect has been noted when the dose of 50 mg/kg is used and the dose of 200 mg/kg has an optimum effect. Therefore, in our experiments, we used the medium effective dose of 100 mg/kg. The tested extracts were administered per os to the animals of the experimental groups in the form of water solutions at a dose of 100 mg/kg once a day 1 h before feeding. The volume of the solution was 0.5 mL/100g of the animal weight. The duration of the treatment depended on the experiment protocol. The dealcoholised liquid extract of *R. carthamoides* was used as a reference remedy at a dose of 5.0 mL/kg according to the same scheme. 

### 4.4. Models, Methods and Experimental Protocols

#### 4.4.1. Acute Emotional Stress

Acute emotional stress was simulated by the method of water immersion (AES) [32]. The animals encaged into a cylinder were up to xiphoid in the water (t = 22 °C) for 30 min. The rats were divided into 5 groups (*n* = 10 per group): 1—intact rats; 2—control animals exposed to acute emotional stress (AES); 3, 4, 5—rats treated with extracts of *R. uniflorum*, *S. centauroides* and *R. carthamoides* respectively against the background of AES. The extract water solutions were preventively administered for 7 days prior to exposure to stress; the last dose was administered 1 h before it. 2 h after exposure to stress the rats were removed from the experiment and the manifestations of the Selye's triad were estimated: the mass of adrenal glands, thymus and spleen as well as the number of destructions in the stomach mucosa. The concentration of TBARS and reduced glutathione, the activity of superoxide dismutase and catalase were evaluated in the blood serum and plasma [20,33,34,35]. In the blood (serum and plasma), the contents of adrenalin, noradrenalin, adrenocorticotropic hormone, corticosterone and aldosterone were evaluated by the method of enzyme linked immunosorbent assay with the use of analyzer DSX (USA) and photometer STATFAX-2100 (USA). The free radical-scavenging ability of the extracts was tested by DPPH^•^ radical scavenging assay [36]; the radical-scavenging activity against ABTS^•+^ radical cation was measured using the Huyut method [37]; the determination of superoxide anion scavenging activity was measured in phenazine methosulphate-nicotinamide adenine dinucleotidenitroblue tetrazolium systems using Ozen method [38]; the chelating activity for Fe^2+^-ions was measured by the *o*-phenanthroline method [39]. The ascorbic acid was used as a reference standard (Sigma-Aldrich). All experiments were repeated three times.

#### 4.4.2. Chronic Emotional Stress

To simulate the chronic emotional stress we used the modified model of chronic emotional stress [40] according to a 4-week protocol. The rats were exposed to different stress-inducing procedures once a day in the morning (from 8 till 10 a.m.) or in the evening (from 4 to 6 p.m.) according to the following scheme:

day 1 of the week—placing of the rats one-by-one on a small elevated, round and dry platform (d 8 cm) located in a bath with cold water at 8–10 °C for 1 h;
day 2—immobilisation of the rats in a small container for 1 h;
day 3—nuchal fold suspension for 5 min.;
day 4—placing of the rats in a cage with the floor awash with cold water for an hour;
day 5—deprivation of water and food from 6 p.m. till 8 a.m.;
day 6—placing of the rats in the home cage with a slope of 45° from 6 p.m. till 8 a.m.;
day 7—no stress.

The stress procedures were carried out in a stochastic mode (alternating the time and type of stressor) for 4 weeks according to the same scheme for each group.

The animals were divided into 5 groups (*n* = 20 per each group): 1—intact rats (Int); 2—control animals subjected to chronic emotional stress; 3, 4, 5—rats treated with *R. uniflorum*, *S. centauroides* and *R. carthamoides* extracts respectively against the background of chronic emotional stress. Four weeks after stress procedures, the rats were tested using such methods as open field and elevated plus maze [41,42], sucrose preference test [40], tail suspension model [43] and forced swimming test [44]. On completing the experiment, 10 rats from each group were decapitated under light ether anaesthesia, the weight of the adrenal glands, thymus and spleen was determined and the brain was taken for morphological studies. As for the rest alive rats (*n* = 10), they were divided into two groups: in 7 rats, cerebral ischemia was induced by occlusion of the left common carotid artery (ischemia); 3 sham-operated rats were used as an additional control (Sham). The rats of the experimental groups continued to receive the tested extracts according to the above scheme. 7 days after ischemia simulation, the rats were removed from the experiment and the specimens of frontal areas of the brain at the level of the front parietal region were taken for histological analyses. After standard histological processing, the specimens were embedded in paraffin, cut into sections 5-μm thick and stained with cresyl violet. The histological and morphometric analyses of brain micropreparations from the CA1 region of the dorsal hippocampus (on an area of 150 × 300 μm) were performed with the use of a light microscope (‘Motic DMW-B1-223’, China) and the program ‘Motic Images, 2000’; the number of unchanged cells, hyperchromic shrunken neurons and neurons with signs of dystrophy were counted [45]. 

The influence of *R. uniflorum* and *S. centauroides* extracts on the effect of GABA-A-receptors antagonists was estimated in two independent series of experiments with the use of an anxiogenic model [46]. In the first series of experiments, the rats were divided into 4 groups: 1—intact rats (sodium chloride); 2—control rats receiving bicuculline; 3 and 4—rats receiving *R. uniflorum* + bicuculline and *S. centauroides* + bicuculline, respectively. In the second series of experiments, the rats were divided into 4 groups: 1—intact rats (sodium chloride); 2—control rats receiving picrotoxin; 3 and 4—rats receiving *R. uniflorum* + picrotoxin and *S. centauroides* + picrotoxin. The *R. uniflorum* and *S. centauroides* extracts were introduced once a day for 5 days, the last dose was introduced 1 h before testing. The GABA-A-receptors antagonists, i.e., bicuculline and picrotoxin were given as a single dose of 2 mg/kg intraperitoneally 30 min before testing. The influence of the tested extracts on the rats' behaviour was estimated by the elevated plus maze test.

### 4.5. Statistical Analysis

The normality of the distribution was assessed by the Shapiro-Wilk test. All data, presented as mean SEM and *p* < 0.05 was considered to be statistically significant. The statistical analysis between groups was carried out by one-way analysis of variance (ANOVA) followed by Bonferroni’s multiple comparison tests.

## 5. Conclusions

*R. uniflorum* and *S. centauroides* extracts have a stress-protective effect, increasing non-specific resistance to emotional stress induced by the single and long-term action of stress factors. In acute emotional stress, the tested extracts inhibited the development of Selye’s triad, while in chronic stress, they decreased the marked manifestations of anxiety-depressive syndrome. The stress-protective effects of *R. uniflorum* and *S. centauroides* extracts as complexes of bioactive substances have a multi-target nature connected with the inhibition of hyperactivation in the central and peripheral stress-promoting systems of the body. *R. uniflorum* and *S. centauroides* extracts have marked neuroprotective properties against stress-induced and ischemic impairment of neurons in the white rat brain cortex. 

## Figures and Tables

**Table 1:** Retention times (*t*_R_), UV- and ESI-MS spectral data, and content of compounds **1**–**14** found in *Rhaponticum uniflorum* and *Serratula centauroides* herb extracts.

No	*t*_R_, min	Compound ^1^	UV, λ_max_, nm	ESI-MS, *m*/*z*	Content in Extract, mg/g ± S.D.	
					*R. uniflorum*	*S. centauroides*
1	2.11	Arbutin ^S^	283	271 [M–H]^−^	n.d.	24.78 ± 0.49
2	5.02	1-*O*-Caffeoylquinic acid ^S^	327	353 [M–H]^−^	n.d.	1.82 ± 0.03
3	5.48	4-*O*-Caffeoylquinic acid ^S^	327	353 [M–H] ^−^	25.11 ± 0.50	4.19 ± 0.08
4	6.52	5-*O*-Caffeoylquinic acid ^S^	327	353 [M–H]^−^	63.59 ± 1.27	40.85 ± 0.82
5	7.18	1,3-Di-*O*-caffeoylquinic acid ^S^	328	515 [M–H]^−^	2.97 ± 0.06	2.16 ± 0.04
6	9.11	20-Hydroxyecdysone ^S^	246	519 [M+K]^+^, 503 [M+Na]^+^, 481 [M+H]^+^, 463 [(M+H)–H_2_O]^+^, 445 [(M+H)–2 × H_2_O]^+^, 427 [(M+H)–3 × H_2_O]^+^, 409 [(M+H)–4 × H_2_O]^+^	17.83 ± 0.35	27.43 ± 0.55
7	9.22	Luteolin-7-*O*-glucuronide ^S^	347	461 [M–H]^−^, 285 [(M–GlcA)–H]^−^	12.18 ± 0.24	n.d.
8	9.42	3,4-Di-*O*-caffeoylquinic acid ^S^	328	515 [M–H]^−^	14.15 ± 0.28	n.d.
9	9.82	Inokosterone ^S^	245	519 [M+K]^+^, 503 [M+Na]^+^, 481 [M+H]^+^, 463 [(M+H)–H_2_O]^+^, 445 [(M+H)–2×H_2_O]^+^, 427 [(M+H)–3×H_2_O]^+^, 409 [(M+H)–4×H_2_O]^+^	n.d.	5.68 ± 0.11
10	9.92	3,5-Di-*O*-caffeoylquinic acid ^S^	328	515 [M–H]^−^	n.d.	n.d.
11	10.18	Apigenin-7-*O*-glucuronide ^S^	337	445 [M–H]^−^, 269 [(M–GlcA)–H]^-^	16.93 ± 0.37	18.27 ± 0.36
12	10.49	Chrysoeriol-7-*O*-glucuronide ^L^	347	475 [M–H]^−^, 299 [(M–GlcA)–H]^-^	n.d.	16.90 ± 0.31
13	10.92	4,5-Di-*O*-caffeoylquinic acid ^S^	328	515 [M–H]^−^	n.d.	1.53 ± 0.03
14	11.28	1,5-Di-*O*-caffeoylquinic acid ^S^	328	515 [M–H]^−^	2.35 ± 0.05	1.93 ± 0.04

^1^ Compound identification was based on a comparison of retention time, UV and MS spectral data with a reference standard (^S^) or interpretation of UV and MS spectral data and comparison with literature data (^L^). n.d.—not detected.

**Table 2:** The effects of *R. uniflorum* and *S. centauroides* extracts on the Selye’s triad and state of antioxidant system in rats exposed to acute emotional stress (AES).

Parameter	Experimental Groups				
	Intact (no AES)	AES + Saline	AES + *R. uniflorum*	AES + *S. centauroides*	AES + *R. carthamoides*
Thymus weight, mg/100g	57.31 ± 5.23	40.54 ± 2.10 *	56.10 ± 3.78 **	55.52 ± 3.61 **	49.50 ± 2.53 **
Adrenal glands weight, mg/100g	16.04 ± 1.08	25.02 ± 2.51 *	20.31 ± 1.66	15.81 ± 1.98 **	16.33 ± 1.62 **
TBARS, µM/L	12.20 ± 1.03	24.71 ± 1.41 *	15.30 ± 0.78 **	16.63 ± 1.37 **	14.30 ± 0.92 **
Reduced glutathione, mM/L	3.12 ± 0.16	0.80 ± 0.73 *	2.33 ± 0.17 **	2.74 ± 0.04 **	1.52 ± 0.08 **
Catalase, U/mL	8.31 ± 0.61	5.92 ± 0.48 *	7.31 ± 0.46 **	7.50 ± 0.11 **	6.51 ± 0.72 **
Superoxide dismutase, U/mL	15.64 ± 1.08	6.21 ± 0.57 *	9.72 ± 0.05 **	11.32 ± 0.79 **	6.84 ± 0.27 **

Data represent as mean ± S.E.M. *—*p* < 0.05 vs. intact group; **—*p* < 0.05 vs. AES group; TBARS —thiobarbituric acid reactive substances.

**Table 3:** Radical-scavenging activity of *R. uniflorum* and *S. centauroides* herb extracts *in vitro*.

Extract, Compound	DPPH˙, IC_50_, µg/mL	ABTS^•+^, IC_50_, µg/mL	O_2_^•-^, IC_50_, µg/mL	FeCA, IC_50_, µg/mL
*R. uniflorum*	22.80 ± 1.32 *	15.50 ± 1.11 *	55.40 ± 4.10 *	546.51 ± 24.20 *
*S. centauroides*	30.12 ± 1.61 *	25.14 ± 1.92 *	77.31 ± 5.22 *	819.20 ± 41.32 *
Ascorbic acid	4.84 ± 0.11	9.11 ± 0.92	101.10 ± 5.14	150.25 ± 10.11

Data represent as mean ± S.E.M. *—*p* < 0.05 vs. ascorbic acid group. DPPH^•^—radical-scavenging activity against 2,2-diphenyl-1-picrylhydrazyl radicals; ABTS^•+^—radical-scavenging activity against 2,2′-azino-bis(3-ethylbenzothiazoline-6-sulfonic acid cation-radicals; O_2_^•-^—radical-scavenging activity against superoxide radicals; FeCA—Fe^2+^-chelating activity.

**Table 4:** The effect of *R. uniflorum* and *S. centauroides* extracts on the content of stress hormones in the blood in rats exposed to acute emotional stress (AES).

Parameter	Experimental Groups				
	Intact (no AES)	AES + Saline	AES + *R. Uniflorum*	AES + *S. Centauroides*	AES + *R. Carthamoides*
Epinephrine, nM	8.51 ± 0.59	37.81 ± 0.35 *	26.50 ± 1.24 **	24.33 ± 0.89 **	31.61 ± 0.51 **
Norepinephrine, nM	64.12 ± 0.27	120.63 ± 4.71	87.71 ± 5.35 **	103.80 ± 3.15	111.32 ± 3.77
ACTH, pg/mL	15.84 ± 1.69	51.03 ± 4.27 *	31.22 ± 0.86 **	37.42 ± 0.69 **	42.60 ± 2.12 **
Corticosterone, nM	44.30 ± 3.74	65.72 ± 3.80 *	50.52 ± 2.45 **	50.61 ± 3.83 **	54.74 ± 4.32
Aldosterone, pg/mL	271.11 ± 10.42	296.10 ± 11.74 *	226.13 ± 12.4 **	278.04 ± 17.6	257.05 ± 16.61

Data represent as mean ± S.E.M. *—*p* < 0.05 vs. intact group; **—*p* < 0.05 vs. AES group. ACTH—adrenocorticotropic hormone.

**Table 5:** The effects of *R. uniflorum* and *S. centauroides* extracts on the behavioural activity of rats in the open field and elevated plus maze tests in chronic emotional stress (CES).

Parameter		Experimental Groups				
		Intact (no CES)	CES + saline	CES + *R. uniflorum*	CES + *S. centauroides*	CES + *R. carthamoides*
Open Field						
Crossing		29.91 ± 3.01	17.93 ± 1.44 *	27.90 ± 4.02 **	31.85 ± 4.80 **	24.33 ± 2.31
Rearing		6.10 ± 0.82	3.40 ± 0.05 *	14.35 ± 0.81 **	12.63 ± 2.50 **	4.12 ± 0.31
Peeking in the holes		4.05 ± 0.16	0.61 ± 0.01 *	2.32 ± 0.13 **	3.10 ± 0.08 **	2.55 ± 0.17 **
Boli		1.33 ± 0.10	2.21 ± 0.12 *	2.10 ± 0.14	1.53 ± 0.15 **	1.84 ± 0.10 **
Elevated plus maze test						
Number of entries	open arm	1.90 ± 0.27	0.25 ± 0.13 *	1.75 ± 0.20 **	2.11 ± 0.41 **	0.53 ± 0.12 **
	closed arm	2.83 ± 0.34	0.72 ± 0.21 *	1.84 ± 0.12 **	2.20 ± 0.25 **	2.21 ± 0.04 **
Duration of stay	open arm	70.32 ± 5.61	13.21 ± 2.12 *	39.11 ± 5.63 **	48.71 ± 2.91 **	22.80 ± 2.05 **
	closed arm	216.1 ± 30.10	280.2 ± 5.60*	239.1 ± 15.05 **	250.0 ± 10.04 **	259.2 ± 6.10 **

Data represent as mean ± S.E.M. *—*p* < 0.05 vs. intact group; **—*p* < 0.05 vs. CES group.

**Table 6:** The effects of *R. uniflorum* and *S. centauroides* extracts on the contents of different neurons in the dorsal hippocampus (CA1) in rats subjected to chronic emotional stress (CES) and ischemia.

Experimental Groups	Hippocampus Neurons (CA1), LH (%)			Hippocampus Neurons (Ca1), RH (%)		
	Hyperchromic	Dystrophic	Normal	Hyperchromic	Dystrophic	Normal
Intact	1.32 ± 0.41	2.33 ± 0.70	96.71 ± 0.62	1.44 ± 0.74	2.40 ± 0.64	96.25 ± 8.12
Sham	3.50 ± 0.51	17.51 ± 1.53	79.03 ± 3.01	4.81 ± 0.50	12.10 ± 0.91	82.13 ± 1.71
Control (CES + ischemia)	64.11 ± 8.52 *	15.80 ± 2.04	20.12 ± 4.11 *	21.41 ± 2.02 *	17.43 ± 3.25	60.81 ± 8.15 *
CES + ischemia + *R. uniflorum*	44.12 ± 8.87 **	25.92 ± 5.33 **	29.14 ± 4.05 **	23.06 ± 2.41 **	5.44 ± 3.21	71.53 ± 10.31
CES + ischemia + *S. centauroides*	41.11 ± 9.80 **	21.94 ± 2.37	37.10 ± 5.06 **	13.42 ± 2.04 **	17.41 ± 3.24	69.22 ± 10.21
CES + ischemia + *R. carthamoides*	39.20 ± 7.10 **	19.90 ± 4.18 **	40.95 ± 3.72 **	15.51 ± 3.01	2.85 ± 4.72 **	81.70 ± 9.90

Data represent as mean ± S.E.M. *—*p* < 0.05 vs. intact and sham groups; **—*p* < 0.05 vs. CES + ischemia; LH—left hemisphere, RH—right hemisphere, Sham—sham operated rats (additional control).

**Table 7:** Regression equations, correlation coefficients (*r*^2^), standard deviation (*S*_YX_), limits of detection (LOD), limits of quantification (LOQ) and linear ranges for 13 reference standards.

Compound	Ionization ^a^	Optimised MRM Transitions, m/z		CE ^b^ (eV)	Regression Equation ^c^		*r* ^2^	*S*_YX_	LOD/ LOQ (µg/mL)	Linear Range (µg/mL)
		Precursor	Quantifier		*a*	*b*∙10^6^				
Arbutin	N	271	107	−10	0.1756	−0.0144	0.9967	3.01∙10^−2^	0.56/1.71	2.00–850.0
1-*O*-Caffeoylquinic acid	N	353	127	−15	2.5394	−1.2360	0.9994	0.45∙10^−2^	0.006/0.02	0.02–300.0
4-*O*-Caffeoylquinic acid	N	353	173	−15	2.7365	−1.0690	0.9996	0.51∙10^−2^	0.006/0.02	0.02–300.0
5-*O*-Caffeoylquinic acid	N	353	165	−15	2.9021	−1.4184	0.9998	0.39∙10^−2^	0.004/0.01	0.02–300.0
1,3-*O*-Caffeoylquinic acid	N	515	179	−15	2.4176	−1.5647	0.9994	0.40∙10^−2^	0.005/0.02	0.02–300.0
20-Hydroxyecdysone	P	481	463	+20	1.6705	−0.4374	0.9988	12.79∙10^−2^	0.25/0.77	0.8–100.0
Luteolin-7-*O*-glucuronide	N	461	285	−20	1.4412	−0.6211	0.9930	11.25∙10^−2^	0.26/0.78	0.80–100.0
3,4-Di-*O*-caffeoylquinic acid	N	515	191	−15	1.1541	−0.4691	0.9987	1.06∙10^−2^	0.03/0.10	0.10–350.0
3,5-Di-*O*-caffeoylquinic acid	N	515	173	−20	0.9562	−0.0521	0.9971	7.79∙10^−2^	0.27/0.82	0.90–100.0
Apigenin-7-*O*-glucuronide	N	445	269	−15	7.064	−1.533	0.9992	1.92∙10^−2^	0.009/0.03	0.03–500.0
Chrysoeriol-7-*O*-glucoside	N	461	299	−20	2.0384	−0.3640	0.9975	2.02∙10^−2^	0.03/0.10	0.10–350.0
4,5-Di-*O*-caffeoylquinic acid	N	515	203	−15	3.6748	−0.7069	0.9987	0.90∙10^−2^	0.008/0.02	0.02–400.0
1,5-Di-*O*-caffeoylquinic acid	N	515	191	−20	1.4689	−0.3641	0.9990	5.69∙10^−2^	0.12/0.38	0.40–400.0

^a^ Ionization mode: N—negative; P—positive; ^b^ CE—collision energy; ^c^ Regression equation: *y* = *a*∙*x* + *b*.

## Data Availability

Data is contained within the article.
